# Extensive necrosis of visceral melanoma metastases after immunotherapy

**DOI:** 10.1186/1477-7819-6-30

**Published:** 2008-03-04

**Authors:** David Stoeter, Nicola de Liguori Carino, Ernest Marshall, Graeme J Poston, Andrew Wu

**Affiliations:** 1 The Department of Hepatobiliary Surgery, Aintree University Hospital NHS Foundation Trust, Lower Lane, Fazakerley, Liverpool, L9 7AL, UK; 2 The Clatterbridge Centre for Oncology NHS Foundation Trust, Clatterbridge Road, Bebington, Wirral, CH63 4JY, UK

## Abstract

**Background:**

The prognosis for metastatic melanoma remains poor even with traditional decarbazine or interferon therapy. 5-year survival is markedly higher amongst patients undergoing metastatectomy. Unfortunately not all are suitable for metastatectomy. Alternative agents for systemic therapy have, to date, offered no greater rates of survival beyond traditional therapy. A toll-like receptor 9 agonist, PF-3512676 (formerly known as CPG 7909) is currently being evaluated for its potential.

**Case presentation:**

We present the case of a 54-year-old Caucasian male with completely resected metastatic cutaneous melanoma after immunotherapy. The patient initially progressed during adjuvant high-dose interferon, with metastases to the liver, spleen, and pelvic lymph nodes. During an 18-month treatment period with PF-3512676 (formerly known as CPG 7909), a synthetic cytosine-phosphorothioate-guanine rich oligodeoxynucleotide, slow radiologic disease progression was demonstrated at the original disease sites. Subsequent excision of splenic and pelvic nodal metastases was performed, followed by resection of the liver metastases. Histologic examination of both hepatic and splenic melanoma metastases showed extensive necrosis. Subsequent disease-free status was demonstrated by serial positron emission tomography (PET).

**Conclusion:**

Existing evidence from phase I/II trials suggests systemic treatment with PF-3512676 is capable of provoking a strong tumor-specific immune response and may account for the prolonged tumor control in this instance.

## Background

Metastasis is by far the most common cause of death in patients with malignant melanoma, with estimated rates of cutaneous malignant melanoma metastasis of approximately 14% [1]. Furthermore, only about 7% of metastatic melanoma cases are suitable for metastasectomy. In general, less than 6% of patients with metastatic disease survive up to 5 years, but in those undergoing complete resection, survival approaches 25% at 5 years [2,3]. In patients unsuitable for surgical intervention, systemic therapy with dacarbazine remains the standard of care with few long-term survivors and a median survival of only 6 months [4]. A number of novel agents are currently undergoing evaluation with the hope of improving outcomes beyond traditional therapy [5,6]. The case report we present describes unique radiological and histopathological responses to one particular new agent: PF-3512676.

## Case presentation

**Figure 1:**
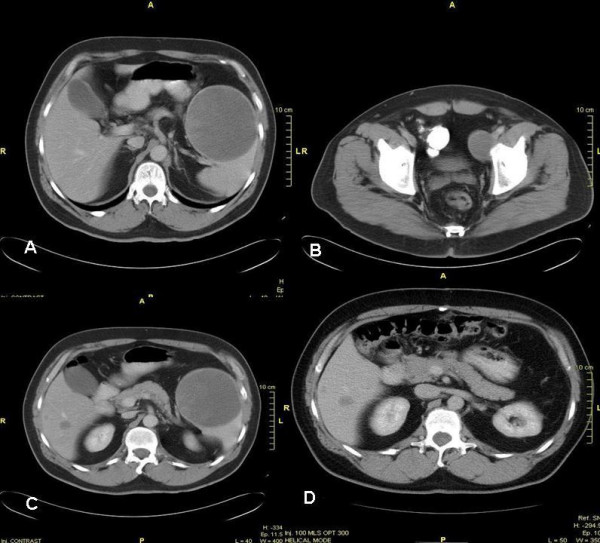
**Computed tomography (CT) of the abdomen and pelvis**. CT scanning immediately before initial pelvic tumor debulking at 46 months after presentation shows A). Large splenic metastasis (100.3 mm), B). Metastasis to the left iliac vein lymph nodes (41.3 mm), and C). A slightly lower view of the splenic metastasis showing the 2 liver lesions. D). CT of the abdomen, post-splenectomy and pelvic tumor debulking, and pre-liver resection (2 liver metastases 20 mm and 15 mm in segments V and VI) at 51 months.

A 54-year-old Caucasian male presented with a 1.5 mm Breslow malignant melanoma on his left shin in February 2002 and was managed with wide local excision. At 16 months follow-up, recurrence in the left inguinal lymph nodes was detected. The patient underwent groin dissection, and all 8 lymph nodes showed either partial or complete replacement by metastatic melanoma on histology with extracapsular extension. No necrosis was seen in either the skin or the lymph node specimens at this stage. Surgery was followed by adjuvant high-dose interferon-α2a therapy, consisting of 1 month of intravenous interferon 5 days a week followed by 11 months of subcutaneous lower-dose interferon 3 days a week. Progression was documented at 6 months by computed tomography (CT), which confirmed a large metastasis in the spleen, left iliac lymphadenopathy, and 2metastases in the liver (Figure 1).

The patient enrolled into a randomized phase II clinical trial in which he was treated with the toll-like receptor 9 agonist PF-3512676 (formerly known as CPG 7909), a synthetic cytosine-phosphorothioate-guanine rich oligodeoxynucleotide. PF-3512676 induces activation of plasmacytoid dendritic cells and enhances cell surface expression of costimulatory molecules (eg, B7) and is hypothesized to promote an antitumor immune response by enhancing antigen presentation to T cells and promoting proliferation of antigen-specific cytotoxic T lymphocytes. CT scanning was performed during the study initially 6 weekly for 6 months, then at 8 weekly intervals. The patient achieved stable disease over a 20-month period. Thereafter, disease progression, according to RECIST radiological parameters [7], was documented at all disease sites. It is worth noting that during the therapy period, the patient experienced grade 1cutaneous reaction (minor erythema) at the site of the excised primary lesion, the left shin. Figure 1A–C shows the 100.3 mm splenic metastasis (Figure 1A), the 41.3 mm pelvic metastases along the left iliac vein lymph node chain (Figure 1B), and the 2 metastatic liver lesions (Figure 1C and 1D) shortly after completion of the PF-3512676 treatment period. In view of the indolent disease biology and the absence of new metastatic lesions during treatment, tumor metastasectomy was undertaken. Initial left iliac lymphadenectomy and splenectomy was followed by adjuvant pelvic radiotherapy. During a second procedure, the patient underwent staging laparoscopy followed by liver resection of segments V and VI with cholecystectomy. Surgical clearance was confirmed by whole body PET scanning at 1 month and 6 months after liver resection, indicating no disease activity. Following up imaging is currently being performed with 3 monthly alternating CT and PET scanning.

### Histology findings

**Figure 2:**
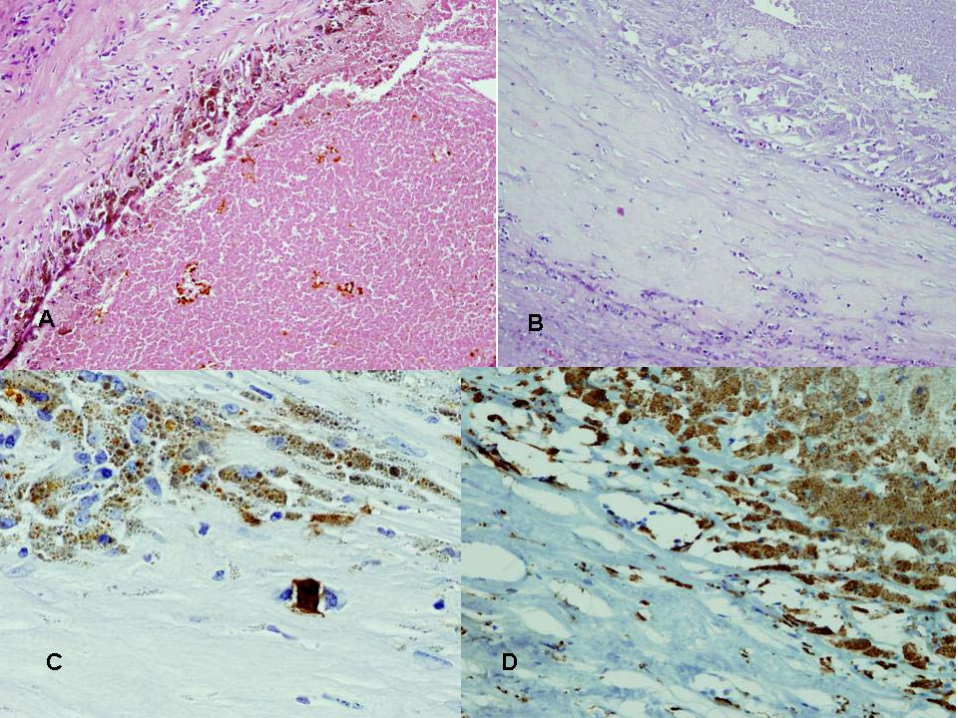
**Photomicrograph of liver metastases**. A). Liver metastasis showing almost complete necrosis within a fibrous capsule. Cells adjacent to the capsule contain a brown pigment. B). The pigment disappears in melanin bleach preparation. C). Very few cells stained positive for S100. In this section, only 1 positive cell was observed, staining a homogeneous dark brown color compared with the dotted granular brown appearance of neighboring cells. D). The majority of the pericapsular cells containing melanin stained positive for CD68, indicating that they are macrophages.

Macroscopic examination of the liver lesions revealed a dark brown nodule (25 × 20 × 15 mm) comprised of 2 adjoining lesions reaching the liver capsule but remaining clear of the resection margin. Sections throughout the specimen revealed an almost entirely necrotic nodule with a well-demarcated fibrous capsule (Figure 2). Many of the cells within the inner margin of the capsule contained a granular brown pigment showing no reaction in Perl's preparation. However, because of loss of color when placed in a melanin bleach preparation, the pigment was identified as melanin. The majority of these pigmented cells tested positive for CD68, indicating that they were macrophages. In contrast, a very few number of cells tested positive for S100, indicating that these cells were melanoma. Hence, the vast majority of these pigmented cells were macrophages, with only a small number identified as melanoma cells.

The spleen specimen also showed a similar histologic pattern, with almost the entire parenchyma replaced by friable necrotic tissue. Moreover, sections contained large areas of necrosis with admixed melanoma cells. In addition, the left iliac specimen was largely hemorrhagic with blood clots, fibroadipose tissue, and deposits of viable malignant melanoma.

## Discussion

This case report illustrates the potential role for metastasectomy in selected cases of metastatic melanoma, despite multiple disease sites. The history of this patient followed a remarkably indolent course on PF-3512676 therapy, even with early recurrence on high-dose interferon. Although progressive disease was documented according to RECIST criteria, there was in fact minor enlargement of the known disease over a number of months. The pathology suggests an inflammatory response to account for the latter, but it remains unclear whether the history was a consequence of indolent biology or therapy.

Malignant melanoma is known to be 1 of 3 types of malignancies with a particular tendency to spontaneously regress. The other 2 types of malignancies are neuroblastoma and renal cell carcinoma [8]. Malignant melanoma accounts for 11% of all reported cases of spontaneous cancer regression but only contributes 1.8% of the total incidence of all types of cancer. However, most of these regressions are partial and rarely occur in metastatic cases (0.22% to 0.27% of all malignant melanoma cases) [8-13]. Regression of visceral metastatic melanoma compared with local cutaneous, subcutaneous, and lymph node spread is exceptionally rare [11-13]. In addition, spontaneous regression of visceral melanoma metastases appears to be short-lived, with only 1 case in the literature demonstrating long-term regression [14]. The observations in this patient suggest that immunotherapy with PF-3512676 may have contributed to the necrosis of metastatic lesions.

The traditional approach to metastatic melanoma remains systemic therapy, with no consistent survival advantage reported with multiagent therapy. Immune therapy-based treatment may induce more durable remissions than standard chemotherapy in highly selected patients; however, a survival advantage has not been confirmed from randomized clinical trials [15]. Even the more recent, experimental agents (IL-2, IL-12, Granulocyte Macrophage-Colony Stimulating Factor and antigen vaccines as well as agents aimed at inhibiting oncogene activity) have showed no clinical benefit over decarbazine in Phase III trials in terms of progression-free survival [16,17]. Success of immunotherapy has been limited by the difficult challenge of provoking an adequate immune response while limiting toxicity. The more recent approaches, aimed at directing the immune response to the tumor cells alone, involve using tumor antigens to produce vaccines. This approach has been applied to Bcell lymphomas and melanomas. However, many vaccines have been proven to be poorly immunogenic and this appears to be due, in part at least, to the immune-dampening effect of the tumor environment. Certain factors have been found to inhibit (e.g. Tregs) or prevent the maturation of an adequate anti-tumor immune response [17]. As such, the search for suitable vaccine adjuvants has been ongoing.

In this case study, a toll-like receptor 9 agonist (PF-3512676) composed of cytosine-phosphorothioate-guanine (CpG)-rich oligodeoxynucleotides was the treatment used. These nucleotides act analogously to bacterial DNA by directly activating plasmacytoid dendritic cells and plasma cells and inducing both T- and B-cell responses as well as cytokine (e.g. interferon-α) production [18,19]. These CpG-rich oligodeoxynucleotides have exhibited evidence of augmenting immune responses to both tumor vaccines and interferon [15] and are currently being evaluated in clinical trials in refractory/relapsing breast cancer, advanced small cell lung cancer, and melanoma [19].

A recent phase I trial, in 8 patients with advanced melanoma, of PF-3512676 with melanoma antigen (Melan A26) compared levels of melanoma-specific cytotoxic T-cells generated by the PF-3512676 vaccine with another vaccine that had demonstrated some success in mice but minimal effect in humans [18]. The PF-3512676 vaccine generated a 43-fold increase in melanoma-specific T-cells compared with prevaccination. This increase was 23 times the level observed with the other vaccine. Reported side effects experienced by the majority of patients included transient malaise, nausea, myalgia, arthralgia, headaches, and fatigue. All patients also experienced inflammation at the injection site on each vaccination. In another study, the combination of interferon α and CpG oligodeoxynucleotides was shown to have additive antitumor activity in murine models of melanoma [20]. Results indicated that CpG oligodeoxynucleotides appeared to greatly enhance tumor infiltration compared with interferon.

Another placebo-controlled Phase II trial looked at the effect of PF3512676 on the immunological response at the sentinel lymph node in patients with early stage melanoma. Results showed a significant increase in the number of mature dendritic cells capable of stimulating T-cells and a reduction in Treg immunosuppression [21].

Most of the phase II trials to date have been directed more at investigating the effects of PF-3512676 on the host immune system and less on actual clinical outcomes of patients, in a bid to first elicit the molecular and cellular mechanisms of action to provide a biological basis for its use in humans. However one recent phase II trial of single-agent PF-3512676 for 24 weeks in patients (N = 20) with metastatic melanoma was performed with an analysis of clinical response as measured by the RECIST parameters [22]. Two patients experienced a partial response, and three patients had stable disease for 8 weeks, and then progressed. The remainder progressed throughout the trial. Responses were observed in lung, skin, and soft tissue. Treatment duration and follow up was much shorter than that performed in our patient. Patient numbers were also small.

## Conclusion

The case we present here appears to be the only such case in the literature reporting evidence of necrosis of extensive visceral metastases in response to this new form of immunotherapy. The data above suggest that PF-3512676 is capable of inducing a strong tumor antigen-specific T-cell response when used with tumor vaccines and can induce marked necrosis in melanoma metastases when used as a single agent. An ongoing phaseI/II clinical trial using 2 different doses of PF-3512676 has indicated no dose-related differences in side effect profiles to date. However, the balance between tumor response and toxicity requires further attention [23]. The majority of research on CpG oligodeoxynucleotides to date has focused on eliciting cellular mechanisms in animal models. Results from current and new clinical trials in humans are awaited to evaluate clinical responses.

## Competing interests

The author(s) declare that they have no competing interests.

## Authors' contributions

DS the compilation and writing of the article, NDLC concept and supervision of the writing of the article, EM contributions to the content of the article and revisions of the final manuscript, AW the operating Surgeon and overseer of the writing of the article.

All authors read and approved the manuscript.
